# Detection of *Mycoplasma agalactiae* in Ticks (*Rhipicephalus bursa*) Collected by Sheep and Goats in Sicily (South-Italy), Endemic Area for Contagious Agalactia

**DOI:** 10.3390/microorganisms9112312

**Published:** 2021-11-08

**Authors:** Paola Galluzzo, Sergio Migliore, Roberto Puleio, Lucia Galuppo, Francesco La Russa, Valeria Blanda, Serena Tumino, Alessandra Torina, Anne Ridley, Guido R. Loria

**Affiliations:** 1 OIE Reference Laboratory for Contagious Agalactia, Istituto Zooprofilattico Sperimentale della Sicilia, Via Gino Marinuzzi, 3, 90129 Palermo, Italy; paola.galluzzo@izssicilia.it (P.G.); roberto.puleio@izssicilia.it (R.P.); galuppolucia@gmail.com (L.G.); serena.tumino@unict.it (S.T.); guidoruggero.loria@izssicilia.it (G.R.L.); 2 Dipartimento Scienze e Tecnologie Biologiche, Chimiche e Farmaceutiche, University of Palermo, Viale delle Scienze, 90128 Palermo, Italy; 3 Entomology Laboratory, Istituto Zooprofilattico Sperimentale della Sicilia, Via Gino Marinuzzi, 3, 90129 Palermo, Italy; francesco.larussa@izssicilia.it (F.L.R.); valeria.blanda@izssicilia.it (V.B.); alessandra.torina@izssicilia.it (A.T.); 4 Department of Bacteriology, OIE Reference Centre for Contagious Agalactia, Animal and Plant Health Agency, Addlestone KT15 3NB, Surrey, UK; Anne.Ridley@apha.gov.uk

**Keywords:** contagious agalactia, *Mycoplasma agalactiae*, ticks, sheep and goats

## Abstract

The aim of this preliminary study was to investigate the presence of *Mycoplasma agalactiae* (*Ma*) or other Contagious Agalactia (CA) causative organisms, in hard ticks infesting milking sheep and goats in endemic areas for CA in Sicily (South-Italy). Although there is accumulating evidence to support the role of ticks in the transmission of blood-borne haemoplasmas, information regarding their role in the transmission of CA, remains scarce. Ticks (*n* = 152) were collected from 25 lactating sheep and goats from three farms with previous outbreaks of CA. Microbiological and biomolecular, as well as serological analysis were performed on milk, tick, and serum samples, respectively. *Rhipicephalus bursa* species predominated, comprising 84.8% of the sampled ticks. Mycoplasma-like colonies were isolated from 5/56 (8.9%) tick pools and were identified as *Ma* by specific PCR and 16S rRNA gene sequencing. Unexpectedly, the organism was isolated from *R. bursa* ticks recovered only from animals whose milk tested negative for the pathogen. This preliminary demonstration suggests the potential role for ticks to act as a reservoir for the organisms, with potential involvement in the spread and maintenance of CA. Further work is required to determine the location of the organisms within the body of the ticks and to assess transmission potential.

## 1. Introduction

Hematophagous ectoparasites are efficient vectors of the disease. More than 200 different diseases of viral, bacterial, rickettsial, protozoan, and helminth aetiology are known to be transmitted by arthropod vectors, including several diseases of major zoonotic importance [1]. Thirty-six vector-borne diseases were recently highlighted as of particular importance within the European Union [2]. Ticks are obligatory hematophagous ectoparasites and around 900 species have been described [3]. Hard (Ixodidae) and soft (Argasidae) ticks, parasitize their hosts for the blood meal only. In addition, their survival is heavily influenced by environmental conditions as well as by the their capacity to find hosts to perpetuate their life cycle [4].

Hard ticks belonging to the genera *Amblyomma, Dermacentor, Haemophysalis, Hyalomma,* and *Rhipicephalus* frequently parasitize small ruminants. Moreover, in Europe, at least 12 different species have been identified, most commonly *Dermacentor marginatus*, *Haemaphysalis punctata*, *Ixodes ricinus,* and *Rhipicephalus bursa* [5].

In animals, mycoplasmas are typically associated with pneumonia, arthritis, and reproductive disorders, often with a chronic and persistent nature [6]. However, despite their association with the disease for many years, transmission pathways together with many other aspects of mycoplasmosis, remain unclear. Ticks have been proposed as reservoirs or vectors, associated with the transmission of several members of the order of *Mycoplasmatales* [7], with a role for arthropods first confirmed in flea and mite species [8]. Subsequently, several studies have indicated that hematophagous ectoparasites may represent the natural vehicle of the uncultivable blood-borne haemoplasma transmission among cats, including *Mycoplasma haemofelis* [9,10,11,12,13], “*Candidatus* Mycoplasma haemominutum”, and “*Candidatus* Mycoplasma turicensis” [7,14,15,16]. In dogs, *Mycoplasma haemocanis* has been successfully transmitted between dogs via the *Rhipicephalus sanguineus* tick [17,18].

In ruminants, mosquitoes and hematophagous flies have been linked to transmission of the haemoplasmas *Mycoplasma wenyonii* and “*Candidatus* Mycoplasma haemobos” [19]. Although first reported in cattle [20], the latter organism is found in water buffaloes, sheep, goats, red deer, fallow, and roe deer, in which it is associated with a drop in milk production, lower calf birth weight, fever, anorexia, depression, and hematuria [21,22,23,24,25]. Flies, lice (*Linognathus ovillas*), and mosquitos have a proposed minor role in the transmission of another haemotrophic *Mycoplasma ovis* [26]. However, despite one previous demonstration of goat fleas of the order Siphonaptera acting as vectors for the transmission of the Contagious Agalactia (CA) agent *Mycoplasma mycoides capri* (formerly LC), and the reproduction of mycoplasmal polyarthritis with septicaemia in goat kids [8], the association of ticks with the transmission of Mycoplasma species causing OIE listed diseases of small ruminants [25] has not been demonstrated to date.

Contagious Agalactia (CA) is mainly a disease spread in traditional husbandry that affect sheep and goats reared for milk and dairy products. Characterized by monolateral or bilateral mastitis and less frequently keratoconjunctivitis, arthritis, and abortion, it is associated with strong financial losses to breeders, primarily due to high morbidity in sheep populations throughout many regions of the world [27,28,29]. Italy typically reports about 50 outbreaks yearly, mainly notified on Sardinia and Sicily (https://www.oie.int/en/animal-health-in-the-world/the-world-animal-health-information-system/the-oie-da%20wahis) (12 February 2021).

In Sicily (Southern-Italy), the disease is predominately caused by *M. agalactiae*, in goats as well as sheep, where it has been endemic for decades, with the first clinical observations dating back to the 1950s. It was known as “Mal di sito” (site sickness) [28] for the risk, which was well recognized by experienced shepherds and veterinarians, of naïve animals being infected as a result of grazing pasture or surroundings previously frequented by an infected flock. The potential link with ecological/environmental aspects associated with survival outside of the main host and transmission in the absence of close contact remain an important data gap (www.discontools.eu) (22 February 2021).

Tick presence in sheep and goats is commonly observed between Spring and Autumn. However, a role for hematophagous arthropods in maintaining and spreading the disease among flocks and contributing to the maintenance of endemism in the area has not, to our knowledge, been investigated to date.

The purpose of this preliminary study was to investigate whether *M. agalactiae* was present in hard ticks infesting sheep and goats reared on three farms, which had historically experienced outbreaks caused by this pathogen, in endemic areas of Sicily.

## 2. Materials and Methods

### 2.1. Ethical Statement

The study did not involve controls under EU Directive 2010 (2010/63/EU), as the blood samples were collected for the purpose of annual brucellosis monitoring, the milk samples were collected from routine milking, and tick removal was below the threshold of the directive and also improved animal health.

For the purpose of this study, permissions from the farmers were sought in advance for the use of these samples and for the collection of ticks from sheep and goats affected by tick infestation.

### 2.2. Study Design and Sampling

Three different Sicilian dairy farms of sheep and goats were selected for the study. The farms were located in the provinces of Palermo (North-West of Sicily) (farms A and B) and Messina (North-East of Sicily) (farm C), on which *M. agalactiae* disease outbreaks were confirmed in 2017, 2016, and 2018, respectively. Following the initial outbreaks on the farms, all of which practiced traditional husbandry for milk production, the disease has subsequently been clinically controlled by annual vaccination with commercial inactivated vaccines. Despite this, occasional, mild symptoms have been reported in a few lactating animals (<2% of the flock). Therefore, the rationale for investigating these previously notified outbreak farms was related to the likely intermittent shedding, as indicated by the consistent isolation of the pathogen, from a limited number of sheep, when monitored throughout the prolonged post-outbreak study period.

In these locations of Sicily, late spring and early summer represent the main periods of tick activity. In addition, tick attachment to the animals on these farms has been previously noted. Therefore, ticks were collected during visits made to each of the three farms between June and July 2020. Information relating to the farms’ location and flock management are shown in Table 1.

Ticks were randomly collected from 25 lactating sheep and goats that were presented with a substantial number of attached parasites. Using fine surgical forceps, several ticks at various stages of engorgement and irrespective of life stage, were collected from different body sites. In order to monitor for active *M. agalactiae* infection in these hosts, including active excretion of the organism in milk and circulation in blood, milk from both udders, contextually to ticks, was collected during traditional milking, while blood was collected as part of yearly Brucellosis prophylaxis. Together with the ticks, samples were placed in a portable cool box and immediately transferred to the OIE Reference laboratory for Contagious Agalactia at the Istituto Zooprofilattico Sperimentale (IZS) of Sicily in Palermo for *Mycoplasma* spp. detection. Ticks were submitted to the Diagnostic Parasitology Laboratory at IZS for species and stage identification.

### 2.3. Tick Identification

On arrival at the laboratory, ticks were collected and kept alive for a week at room temperature, in order to allow the ectoparasites to cleanse themselves of any ingested blood.

Species, sex, and status identification of ticks followed standard morphological observations [30].

Once morphologically identified, ticks were stored at −20 °C until further examination. Each tick was bathed in 70% ethanol for 5 min [31] and divided lengthwise into two parts in sterile Petri dishes under the stereomicroscope, using sterile forceps and scalpels: One half was screened by cultural and molecular methods, with the remaining half kept in alcohol, pending further investigation. Ticks belonging to the same species and stage, and derived from the same animal, were analyzed in pools. Each pool contained between one and five ticks and was homogenized in 500 μL of Mycoplasma broth before sub-culturing for pathogen isolation and molecular tests.

### 2.4. Microbiological Analysis

For culturing of viable *Mycoplasma* spp., 300 μL of milk or 300 μL of tick homogenate was transferred to a sterile bijoux containing 2.7 mL of Mycoplasma broth medium [32] and incubated at 37 °C in an atmosphere containing 5% CO_2_. After 24 h, the broth culture was passed through a 0.45 μm filter to exclude the growth of other potentially contaminating microorganisms. Following 48 to 72 h of incubation, 10 μL of broth was subcultured on Mycoplasma agar media, then it was monitored every 24 h for up to 7 days for the presence of typical “fried egg” colonies [33].

### 2.5. Serological Analysis

Blood samples were gently centrifuged at 3000× *g* for 10 min to collect serum. Antibodies against *M. agalactiae* were detected using the commercially available ID Screen^®^ *Mycoplasma agalactiae* Indirect ELISA kit (ID Vet, Grabels, France). The test was performed according to the manufacturer’s instructions. As recommended, the samples were considered positive if the sample to positive (SP) ratio was above 60. If the SP ratio was between 50 and 60, the result was considered doubtful, while samples with an SP ratio under 50 were interpreted as negative.

### 2.6. DNA Extraction and PCR Amplification

From the remaining 200 μL from each pool or singular tick homogenate, DNA was extracted using the Quick-DNA Miniprep Plus Kit (Zymo Research, Irvine, California, USA), following the manufacturer’s instructions. To confirm the presence of *M. agalactiae* DNA, a commercial TaqMan real-time PCR was performed. The VetMAX *M. agalactiae* and *M. mycoides* Kit (Thermo Fisher Scientific, Waltham, MA, USA) were used to determine the presence of other agents responsible for CA in goats (mycoplasmas of the *M. mycoides* group) at the same time as *M. agalactiae*. Real-time PCR was performed using the CFX96 Touch Real-Time PCR Detection System (Bio-Rad, Hercules, CA, USA).

### 2.7. DNA sequencing

All of the cultures showing Mycoplasma-like colonies had the DNA extracted, followed by PCR and sequencing to confirm microbiological observations. A semi nested-PCR using general and operon-specific primers was performed to amplify the 16S rRNA gene [34]. The first amplification was performed with the primers U1 (5′-GTTTGATCCTGGCTCAGGCYDAAC-3′)/U8(5′-GAAAGGAGGTRWTCCAYCCSCAC-3′). The second amplification was performed using primers U1/U5 (5′-CTTGTGCGGGTCCCCGTCAATTC-3′), and the other with primers U2 (5′-CCAGACTCCTACGGGAGGCAGC-3′)/U8. Amplicons were purified by the QIAquick Gel Extraction Kit (Qiagen, Hilden, Germany), quantified, and sent for sequencing (BMR Genomics srl., Padova, Italy). The obtained sequences were aligned using the Bioedit software (Tom Hall, Ibis Biosciences, Carlsbad, CA, USA) and analyzed for nucleotide sequence identity. Then, they were compared with the reference strains in the GenBank database using the Basic Local Alignment Search Tool (BLAST). The multiple sequence alignment and maximum likelihood phylogenetic tree construction were carried out using the Mega 11 software and were compared with two *M. agalactiae* (5632 and PG2) and *M. bovis* PG45. The obtained sequences were deposited on the GenBank database (MZ621182, MZ621183, MZ621184, MZ621185, MZ621186).

## 3. Results

### 3.1. Ticks Identification

**Figure 1:**
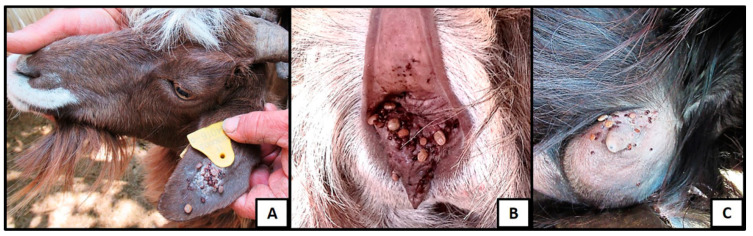
Tick infestations: (**A**) Ear lobe, (**B**) vulva, and (**C**) udder.

A total of 152 ticks were collected from 25 animals from the three different farms. From those, 102 were removed from goats, with a remaining 50 from sheep. The ticks were randomly collected from sheep and goats, corresponding to the areas of hairless skin, including the vulva, perineum, udders, and ears (Figure 1).

All of the ticks were adults and were morphologically identified as *Rhipicephalus bursa* 84.8% (129/152), *Rhipicephalus sanguineus* 13.8% (21/152), *Rhipicephalus turanicus* 0.65% (1/152), and *Rhipicephalus hylusitanium* 0.65% (1/152) (Table 2).

On farm A, only R. bursa was found. The tick species, number, stage, and number of the related pool for each farm are summarized in Table S1.

### 3.2. Microbiological Analysis

The microbiological investigations performed on milk samples revealed that only two of the 25 tested, both from goats belonging to farm A, were positive for the presence of Mycoplasma and confirmed as *M. agalactiae* using the VetMAX *M. agalactiae* and *M. mycoides* real-time PCR. No Mycoplasma-like organisms were recovered from the remaining 23 milk samples. Viable Mycoplasma-like colonies were recovered from five of the 48 pools of ticks following culture (Table 3).

Of these positive pools, two belonged to farm B (no. 8 and no. 12; Supplementary Table S1) and comprised non-engorged female and male ticks, respectively, which had been removed from two different sheep. Three pools were positive from farm C, (no. 15, 16, and 17), comprising non-engorged female and male ticks all recovered from the same goat. Non-viable *Mycoplasma* spp. organisms were isolated from ticks recovered from goats or sheep, belonging to farm A. The overall prevalence at the animal level is 12% (3/25) with 0% in farm A, 18.18% (2/11) in farm B, and 12.5% (1/8) in farm C.

### 3.3. Molecular Analysis

The real-time PCR analysis using the VetMAX^TM^ *M. agalactiae* and *M. mycoides* kit revealed 17 *M. agalactiae* positive pools (30.36%). A further two (3.6%) pools were positive to *M. mycoides,* while eight pools were positive to both pathogens (14.3%).

Real-time PCR performed on the recovered isolates from the five *Mycoplasma* spp. positive tick pools confirmed *M. agalactiae* in each pool (Table 3).

### 3.4. Serological Analysis

Serological analysis for host antibody response to *M. agalactiae* showed high prevalence of specific antibodies among the animals sampled. On farm A, all of the six animals tested were positive (100%), which included the two goats from which *M. agalactiae* was recovered from milk. The prevalence was lower, at 36.4% (4/11) and 87.5% (7/8), in the sheep on farm B and goats on farm C, respectively (Table 3). One of the four serologically positive sheep on farm B (animal ID 4; Supplementary Table S1) and one of the seven goats (animal ID 6) on farm C, were animals from which *M. agalactiae* was recovered from the collected ticks.

### 3.5. DNA Sequencing

**Figure 2:**
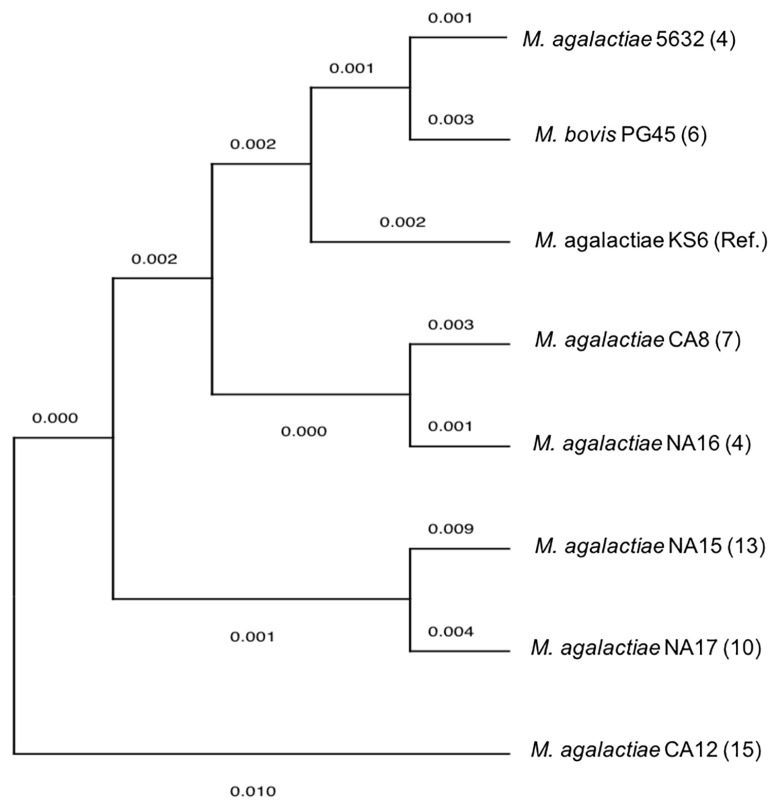
Maximum likelihood Phylogenetic tree of *Mycoplasma agalactiae* isolated from ticks infesting sheep and goats with 16S rRNA gene sequences (1500 bp) using Mega 11. In brackets, the number of SNPs identified in each sequence is compared with the reference sequence (*M. agalactiae* KS6).

The 16S rDNA sequencing analysis was performed on the five strains isolated from ticks. In addition, all five were confirmed as *M. agalactiae* with ≥99% homology within the 1500 bp region (MZ621182 and MZ621183 came from farm B; MZ621184, MZ621185, and MZ621186 came from farm C). A phylogenetic tree was constructed by comparing the 16S rRNA gene sequences obtained from *M. agalactiae* strains isolated from ticks with those present in the database of the most similar strains (Figure 2).

## 4. Discussion

In this first field study on extensive Mediterranean sheep and goat farms, we report for the first time, the identification of *M. agalactiae* in the tick populations feeding on sheep and goats in endemic areas for CA in Sicily. The organism was isolated from five of 56 tick pools sampled from 25 animals, an overall prevalence of 8.9% and 12% at animal level. Despite the fact that positive ticks were collected from different species, sheep in farm B and goats in farm C, the prevalence at animal level in the two farms was similar and further studies on higher consistencies in order to attribute statistical significance are needed.

The five infected pools all comprised *R. bursa*. In addition, this tick species accounted for almost 85% of the ticks collected from the ear lobes, udder, and vulva of sampled sheep and goats, suggesting predominance of this species in small ruminant livestock infestations in Sicily in June and July of 2020, when the study took place. Sicily represents an ideal ecosystem for the study of ticks and the prevalence of tick-borne pathogens [35]. The prevalence of *R. bursa* in the observed infestations of sheep and goats was not unexpected, as this tick is considered a major ectoparasite of sheep in Sicily and the Mediterranean basin [36,37], which spreads in hilly, marginal areas, grassy slopes or semi-desert environments [38]. Tick seasonality in Sicily is not dissimilar in its timing to the seasonal variation of outbreaks of Mycoplasma mastitis in sheep and goats. This tick is already a well-recognized vector of several pathogens in ruminant livestock, including *Babesia ovis* [30] and *Coxiella burnetii* [38], with *Theileria* spp., *Anaplasma marginale*, *Anaplasma ovis,* and *Ehrlichia canis* also transmitted by *R. bursa* [39,40,41,42].

The ticks described in this study were infesting sheep and goat milking herds on three Sicilian farms, on which *M. agalactiae* infection had been previously confirmed and notified. Although these farms have experienced recurrence of mild clinical signs of CA in resident sheep and goats, with excretion of the organism in milk, the respective host animals were not, at least at the point of sampling, detectably shedding the organism in milk. However, it is well recognized that excretion of the organism in milk is often intermittent, particularly in asymptomatic carriers [28,29].

Specific IgG antibodies were demonstrated. However, it is well known that following infection by *M. agalactiae* the persistence of antibodies could be observed, generally up to 8 months, but for as long as 3 years after the outbreak [27]. Moreover, following the original disease incursions on these farms, the sheep and goats had been regularly vaccinated using an inactivated vaccine, as permitted in the European Union. For all of the three farms, the owners confirmed that vaccination against CA, with commercial inactivated vaccine, has been carried out before the beginning of milking period (September/October) of the previous year. Therefore, 10 months before the field sampling took place. Although expected to boost immunity in the field, these vaccines appear to prevent the occurrence of clinical signs and generally limit excretion of *M. agalactiae* [42,43]. However, in the present study, two of the goats were shedding viable *M. agalactiae* in their milk.

Only *M. agalactiae* was recovered following culture, a finding consistent with organisms that recovered the original outbreaks on these farms and during subsequent monitoring. However, an additional number of tested tick pools, including those consisting of other tick species and comprising engorged ticks, were positive by PCR, typically for both *M. agalactiae* and *M. mycoides*. Infections associated with the “*M. mycoides* cluster” organisms are important in goats in a number of affected countries, with the auricular carriage well studied in Spain, where it often occurs without clinical signs [44]. However, in Sicily, *M. agalactiae* is predominant in sheep and goat farms, with only occasional outbreaks in kids attributable to *M. mycoides* subsp. *capri* [6,45], associated with the importation of goats from other affected regions or countries. Therefore, the finding of *M. mycoides* cluster would be unexpected from the knowledge of animal movements on these farms. No sampling was conducted on the body sites infested by ticks to establish this possibility and should be considered in future work.

It is remarkable that all of the five pools from which *M. agalactiae* was recovered only comprised non-engorged adult males and females. The positive ticks were presumably in the early stages of feeding, which can take around 7 days [46]. DNA was extracted from the entire body (often very small) of the parasites, which had been sterilized in 70% ethanol to determine the presence of *M. agalactiae*. Therefore, attempts were not made to differentiate the salivary glands or head from the rest of the body. Ingestion of infected blood in the mid-gut, is a key microbial entry point and one that determines pathogen colonization and survival in the tick. This cultivation from all of the fed tick pools proved unrewarding, suggesting that any *M. agalactiae* ingested remained at numbers below those needed for successful amplification by culture. It was not possible to substantiate this, as the blood drawn from the sampled animals was not cultured or examined by PCR. Therefore, it is not known whether the organism was circulating systemically in these animals.

Migration to the salivary glands entails surviving ingestion following feeding during an earlier stage of the tick lifecycle. From there, transmission to the next host may be facilitated during the next blood meal. While tick-borne pathogens themselves may be very different, they appear to have evolved similar strategies to infect ticks. The competence of the vector to endure a symbiotic relationship with the pathogen facilitates the multiplication and transmission of the pathogen and is essential as a driver for tick-borne disease [47]. For *B. ovis*, both female and male *R. bursa* ticks are implicated in the transmission of the hemoparasite, with females considered to represent a greater threat due to the transovarial transmission and prolonged feeding periods [48]. Following an observation of the possible association of a different Rhipicephalus tick species, *R. (Boophilus) microplus* with *Ca* M. haemobos in sheep and goats in China, Shi et al. [25] conducted feeding experiments. These demonstrated larvae emergence from positive eggs and suggested that *R. (B.) microplus* ticks have the potential to act as reservoirs for “*Ca* M. haemobos”, with female ticks capable of transmitting “*Ca* M. haemobos” transovarially.

Despite a proposed role for ticks and other arthropods in the transmission of haemoplasmas in domestic animals, a role for ticks and other biting insects as potential reservoirs or vectors for CA agents has not been established to date. Direct contact between healthy and infected animals, often with subclinically—infected healthy carriers, and through manual milking are the most common routes of *M. agalactiae* transmission. However, large scale small ruminant pastoralism favors the persistence of *M. agalactiae* in the environment months after an outbreak of disease [28]. Therefore, environmental factors appear to play an indirect, additional role in pathogen maintenance on farms that have experienced outbreaks of the disease. As early as 1862 in Apulia (Italy), Dinella and Provinziano observed that animals became infected after grazing a pasture previously occupied by an infected flock and called the disease “mal del sito” disease of the place [49]. However, as obligate parasites of cells, mycoplasmas are considered poorly resistant, at least over extended periods, to environmental conditions, particularly solar irradiation and desiccation. Despite this, these pathogens are endemic in Mediterranean countries where climatic conditions ensure high temperatures and sunshine over the spring and summer months, suggesting that other factors are at play which support the spread of CA endemism. Moreover, the occurrence of new outbreaks in formerly disease free areas or the arrival of the infection in previously disease free flocks where there is no documented evidence of restocking or contact with others, has remained unexplained. In the present study, *M. agalactiae* was only successfully cultivated from unfed or partially-fed ticks, which were sterilized before the microbiological examination. It is tempting to speculate that *M. agalactiae* may have been residing in the salivary glands in these ticks. However, further work, including examination of segregated body parts including haemolymph to mitigate against the detection of organisms within the blood meal, is required to investigate this hypothesis. Nevertheless, the finding of viable *M. agalactiae* in these common ticks of ruminants in Sicily, albeit only a small proportion of the pooled samples, suggests a potential role for *R. bursa* and other *Ixodidae* as a reservoir and vector perpetuating CA endemism.

## 5. Conclusions

This preliminary study has demonstrated the carriage of viable *M. agalactiae* by *R. bursa* ticks sampled on milking sheep and goat farms, suggesting a potential role of these ticks as a reservoir or potential vector of the pathogen. The existence of these environmental sources for *M. agalactiae* and their role in transmission and clinical disease warrant further investigation. Further field and laboratory investigations are required to investigate the location of *M. agalactiae* inside the Arthropoda S, but also subsequently to determine their possible active role in vertical transmission through their progeny.

## Figures and Tables

**Table 1:** Farm information and number of animals sampled.

Farm	Locality	Farmed Species (n.)	First Report of CA	Sampled Individuals	Collected Ticks
A	Altofonte (PA *)	Sheep (111) Goat (75)	2017	1 sheep 5 goats	45
B	Borgetto (PA *)	Sheep (171) Goat (2)	2016	11 sheep	41
C	Santa Lucia del Mela (ME **)	Sheep (187) Goat (300)	2018	8 goats	66
Total				12 sheep and 13 goats	152

* PA: Palermo, province North—West of Sicily; ** ME: Messina, province North—East of Sicily.

**Table 2:** Tick species and number collected from the three farms investigated.

Farm	Tick Species	No. of Ticks	Total
A	*R. bursa*	45	45
B	*R. bursa*	39	41
	*R. turanicus*	1	
	*R. hylusitanium*	1	
C	*R. bursa*	45	66
	*R. sanguineus*	21	

**Table 3:** Mycoplasma identifications from the ticks and hosts sampled on the three farms comparing ELISA, cultural isolation, and PCR tests.

Farm	ELISA (No. of Positive/All Samples)	*Mycoplasma* spp. Isolation from Milk (No. of Positive/All Samples)	*Mycoplasma* spp. Isolation from Ticks Pools (No. of Positive/All Samples)	Real-time Results from Tick Pools (No. of Positive/All Pools)		
				*M. agalactiae*	*M. mycoides* group	*M. agalactiae/* *M. mycoides*
A	6/6	2/6 ^a^	0/15	3/15	2/15	0
B	4/11	0/11	2/21 ^a^	6/21	0	3/21
C	7/8	0/8	3/21 ^a^	11/20	0	5/20
Tot.	17/25	2/25	5/56	17 ^b^/56	2 ^b^/56	8 ^b^/56

^a^ Identified as *M. agalactiae* by specific PCR and 16S rRNA gene sequencing. ^b^ Defined using the manufacturer’s recommended cut-off value for positivity, which is Ct < 45.

## Data Availability

Sequences were deposited in GenBank (MZ621182, MZ621183, MZ621184, MZ621185, MZ621186).

## Supplementary Materials

The following are available online at https://www.mdpi.com/article/10.3390/microorganisms9112312/s1. Table S1: Host, tick species, sex, state, and distribution in pools of all the collected samples.
