# Supplementary material for: Detection of Mycoplasma agalactiae in Ticks (Rhipicephalus bursa) Collected by Sheep and Goats in Sicily (South-Italy), Endemic Area for Contagious Agalactia
Source: Microorganisms. 2021 Nov 8;9(11):2312. doi: 10.3390/microorganisms9112312 (PMC8625260; doi:10.3390/microorganisms9112312)
Supplement: Supplementary file 1 [file microorganisms-09-02312-s001.zip › microorganisms-1446924-supplementary.pdf]

microorganisms

Supplementary material

Table S1. Host, tick species, sex, state and distribution in pools of all sample randomly collected.

Animal	Tick species (n.)	Sex	Engorged	Pool	Real time PCR		Isolation
	Farm A				***Ma****	***Mm*****	
Goat 1	*R. bursa* (5)	M	-	1	neg	40	neg
	R. bursa (5)	F	No	2	39	neg	neg
Goat 2	*R. bursa* (4)	M	-	3	neg	neg	neg
	R. bursa (2)	F	No	4	neg	40	neg
	R. bursa (2)	F	Yes	5	39	neg	neg
Goat 3	*R. bursa* (3)	M	-	6	neg	neg	neg
	R. bursa (2)	F	No	7	neg	neg	neg
Goat 4	*R. bursa* (2)	M	-	8	neg	neg	neg
	R. bursa (2)	F	No	9	neg	neg	neg
	R. bursa (2)	F	Yes	10	neg	neg	neg
Goat 5	*R. bursa* (3)	M	-	11	40	neg	neg
	R. bursa (4)	F	No	12	neg	neg	neg
Sheep 1	*R. bursa* (2)	M	-	13	neg	neg	neg
	R. bursa (3)	F	No	14	neg	neg	neg
	R. bursa (4)	F	Yes	15	neg	neg	neg
	Farm B						
Sheep 1	*R. bursa* (3)	M	-	1	neg	neg	neg
	R. bursa (2)	F	No	2	neg	neg	neg
Sheep 2	*R. bursa* (3)	M	-	3	neg	neg	neg
	R. bursa (1)	F	Yes	4	38	neg	neg
Sheep 3	*R. bursa* (3)	M	-	5	neg	neg	neg
	R. bursa (1)	F	No	6	neg	neg	neg
Sheep 4	*R. bursa* (2)	M	-	7	neg	neg	neg

	R. bursa (2)	F	No	8	neg	neg	pos
Sheep 5	*R. bursa (1)*	M	-	9	37	neg	neg
	R. bursa (1)	F	No	10	38	38	neg
Sheep 6	*R. bursa (1)*	M	-	11	38	neg	neg
Sheep 7	*R. bursa (5)*	M	-	12	neg	neg	pos
	R. bursa (2)	F	No	13	38	38	neg
	R. hylusitanium (1)	M	-	14	38	37	neg
Sheep 8	*R. bursa (1)*	F	No	15	neg	neg	neg
	R. bursa (1)	M	-	16	neg	neg	neg
Sheep 9	*R. bursa (1)*	M	-	17	neg	neg	neg
	R. turanicus (1)	M	-	18	neg	neg	neg
	R. bursa (2)	F	No	19	neg	neg	neg
Sheep 10	*R. bursa (2)*	F	No	20	neg	neg	neg
Sheep 11	*R. bursa (5)*	F	No	21	neg	neg	neg
	Farm C						
Goat 1	*R. sanguineus (1)*	F	No	1	neg	neg	neg
	R. bursa (1)	M	-	2	neg	neg	neg
	R. sanguineus (5)	F	Yes	3	neg	neg	neg
Goat 2	*R. bursa (1)*	M	-	4	neg	neg	neg
	R. sanguineus (5)	F	Yes	5	neg	neg	neg
Goat 3	*R. bursa (3)*	M	-	6	neg	neg	neg
	R. bursa (3)	F	No	7	38	neg	neg
Goat 4	*R. bursa (1)*	M	-	8	38	38	neg
	R. sanguineus (2)	M	-	9	39	37	neg
	R. sanguineus (1)	F	No	10	38	38	neg
	R. sanguineus (4)	F	Yes	11	neg	neg	neg
Goat 5	*R. sanguineus (3)*	M	-	12	38	neg	neg

	R. bursa (5)	M	-	13	38	neg	neg
	R. bursa (5)	F	No	14	neg	neg	neg
Goat 6	*R. bursa (5)*	M	-	15	38	neg	pos
	R. bursa (5)	F	No	16	38	neg	pos
	R. bursa (5)	F	No	17	40	neg	pos
Goat 7	*R. bursa (2)*	F	No	18	37	36	neg
Goat 8	*R. bursa (5)*	M	-	19	38	38	neg
	R. bursa (4)	F	Yes	20	neg	neg	neg

**M. agalactiae* ***M. mycoides* group
